# Does a loss of TDP-43 function cause neurodegeneration?

**DOI:** 10.1186/1750-1326-7-27

**Published:** 2012-06-14

**Authors:** Zuo-Shang Xu

**Affiliations:** 1 Department of Biochemistry and Molecular Pharmacology, University of Massachusetts Medical School, 364 Plantation St, 817 LRB, Worcester, MA, 01605, USA

## Abstract

In 2006, TAR-DNA binding protein 43 kDa (TDP-43) was discovered to be in the intracellular aggregates in the degenerating cells in amyotrophic lateral sclerosis (ALS) and frontotemporal lobar degeneration (FTLD), two fatal neurodegenerative diseases [1,2]. ALS causes motor neuron degeneration leading to paralysis [3,4]. FTLD causes neuronal degeneration in the frontal and temporal cortices leading to personality changes and a loss of executive function [5]. The discovery triggered a flurry of research activity that led to the discovery of TDP-43 mutations in ALS patients and the widespread presence of TDP-43 aggregates in numerous neurodegenerative diseases. A key question regarding the role of TDP-43 is whether it causes neurotoxicity by a gain of function or a loss of function. The gain-of-function hypothesis has received much attention primarily based on the striking neurodegenerative phenotypes in numerous TDP-43-overexpression models. In this review, I will draw attention to the loss-of-function hypothesis, which postulates that mutant TDP-43 causes neurodegeneration by a loss of function, and in addition, by exerting a dominant-negative effect on the wild-type TDP-43 allele. Furthermore, I will discuss how a loss of function can cause neurodegeneration in patients where TDP-43 is not mutated, review the literature in model systems to discuss how the current data support the loss-of-function mechanism and highlight some key questions for testing this hypothesis in the future.

## Introduction

Amyotrophic lateral sclerosis (ALS) is a disorder where progressive degeneration of large motor neurons in the spinal cord and cerebral cortex leads to paralysis and death [3,4]. Frontotemporal lobar degeneration (FTLD) causes degeneration of neurons in frontal and temporal cortices, leading to deterioration of executive, cognitive and social functions, as well as loss of emotional control [5]. Although clinically distinct, a significant overlap exists between these two diseases in the patient population, resulting in a continuous spectrum ranging from patients with one disease at either end and patients with varying degrees of both diseases in the middle [6,7]. Recent genetic data has reaffirmed the connection between these two diseases. Some genetic mutations cause one disease but rarely the other, e.g. SOD1, FUS and TDP-43 for ALS, and tau, progranulin and CHMP2B for FTLD. Other mutations cause either or both diseases in the same patient or family, e.g. ubiquilin 2 and C9ORF72. In a significant population of patients (~95 % ALS and ~50 % FTLD), TDP-43 positive intracellular inclusions are present in the CNS even though the TDP-43 gene is not mutated [8-11], raising the question of how wild-type TDP-43 is involved in the pathogenesis of these cases.

TDP-43 is a RNA binding protein containing two RNA-recognition motifs (RRM), a nuclear localization signal (NLS) and a nuclear export signal (NES) [12]. The protein is normally concentrated in the nucleus but also shuttles back and forth between the nucleus and cytoplasm [13]. TDP-43 is a global regulator of gene expression and is involved in regulation of transcription and multiple aspects of RNA processing and functioning, including splicing, stability, transport, translation and microRNA maturation [14-17]. TDP-43 interacts with many proteins and RNAs and functions in multi-protein/RNA complexes [18-21]. TDP-43 maintains its protein expression at a constant level within a tight range by auto-feedback mechanisms, which involve TDP-43 binding to its own 3’ untranslated region [15,22]. Overexpression of TDP-43 leads to down-regulation of the endogenous TDP-43 [23,24], and blocking expression of one allele leads to a compensatory increase in the expression of the other allele [25-27]. The tight regulation of TDP-43 levels is suggestive of its crucial role in the functioning of multi-protein/RNA complexes, where maintaining a certain stoichiometry between TDP-43 and the other components may be critical.

Because mutations in TDP-43 lead to ALS, a causal role of TDP-43 for neurodegeneration is firmly established [12,28,29]. Therefore, understanding how the mutants cause neurodegeneration offers a convenient entry point for exploring how TDP-43 plays this role. The first question is whether a gain, a loss of function or a dominant-negative effect mediates neurotoxicity. A resolution to this question is of critical importance because it sets the direction of further research on the disease mechanism and on the design of therapeutic strategies. To answer this question, model systems of both gain or loss of function must be employed (Table 1). Gain-of-function models are usually achieved by gene overexpression and loss-of-function models by gene knockout or knockdown. Based on the phenotypic readouts, the mechanism whereby the mutants cause neurodegeneration can be deduced (Table 1).

**Table 1:** Assay for disease mechanism using transgenic animals

**Transgene expression**	**Disease mechanism**			**TDP-43**		
	GF	LF	DN	Fly(a)	Fish(b)	Rod.(c)
OE mutant	+	**-**	+	+	+	+
OE WT	−/+	**-**	−/+	+	+	+
KO or KD	**-**	+	+	+	+	?

OE = overexpression; KO = knockout; KD = knockdown;

GF = Gain of function; DN = Dominant negative; LF = Loss of function

Rod. = rodents; + = positive for neurological phenotype

- = negative for neurological phenotype; ? = remains to be determined

See the text for detailed explanation and references for the models.

(a) reference [30-34]; (b) reference [35]

(c) reference [12,23-27,36-43]

A gain-of-function (Table 1, GF column) mechanism includes two scenarios: first, the mutant gene gains a novel toxic activity that is independent of the normal function of the gene, and second, the mutant becomes hyperactive in one of its normal functions leading to toxicity. In the first scenario, overexpression of the mutant gene, but not the wild type, will cause the disease phenotype. In the second scenario, overexpression of either the mutant or wild-type gene will cause the disease phenotype. In both gain-of-function scenarios, knockout or knockdown of the gene is not expected to cause the disease phenotype.

A loss of function (haploinsufficiency; Table 1, LF column) means that the mutant gene has no function or a reduced function but does not interfere with the function of the wild-type allele. In this scenario, neither overexpression of the mutant nor the wild type is expected to cause the disease phenotype. But knockout or knockdown reproduces the loss of function, and therefore, is expected to generate the disease phenotype.

A dominant-negative mechanism (Table 1, DN column) denotes the condition where the mutant allele is dysfunctional and inhibitory to the function of the wild-type allele. In this scenario, overexpression of the mutant gene is expected to cause the disease phenotype because it dominant-negatively inhibits the function of the endogenous wild-type protein. On the other hand, overexpression of the wild type is generally not expected to generate the disease phenotype because the wild-type gene can function normally and does not inhibit the function of the normal endogenous allele. However, there are exceptions under certain circumstances, for example, if the protein functions in a multi-protein complex (see details below). Knockout or knockdown of the gene is expected to reproduce the disease phenotype because this reduces the function of the wild-type gene. Thus, in model systems, the dominant-negative mechanism can display characteristics of both a gain and a loss of function—it is a loss of function in essence, yet its effect can dominate over the endogenous wild-type allele.

In the case of TDP-43, an abundance of gain-of-function models have been generated in various species, including worm, fly, fish and rodents [12]. In all models with rare exceptions, a consistent finding is that overexpression of both mutant and wild type TDP-43 can cause a neurodegenerative phenotype (Table 1, TDP-43 columns), thus supporting a gain-of-function mechanism and a potential overactivation of TDP-43 in the mutants [12]. Loss-of-function models have also been generated in non-mammalian species and all except the worm showed neurological and neurodegenerative phenotypes [30-33,35,44]. The difference between worm and the other species may reflect some species difference, since TDP-43 is dispensable for survival in the worm but not so in other species. In general, the degenerative phenotypes in the loss-of-function models appear less overwhelming than the overexpression models and are often difficult to separate from the developmental effects stemming from a lack of TDP-43 function. Importantly, there is a lack of evidence in mammalian models that a loss of TDP-43 function causes neurodegeneration. This is largely due to the failure in generating such a model using a gene knockout approach [25-27,36]. As a result, the current literature leans towards a gain-of-function mechanism as far as the role of TDP-43 in neurodegeneration is concerned.

Yet despite the preponderance of evidence for the gain-of-function mechanism, it has not been sufficient to rule out the loss-of-function mechanism, because the gain-of-function mechanism does not explain well a phenomenon that is consistently observed in numerous pathological studies, i.e. the nuclear clearance of TDP-43 that accompanies the presence of TDP-43 intracellular aggregates [1,2,45]. The question whether the depletion of TDP-43 in the nucleus is consequential in the pathogenesis remains unanswered. In addition, although the aggregates in the cytoplasm may generate gain-of-function type of toxicity, it is also conceivable that the aggregation of TDP-43 renders TDP-43 non-functional, and as such, causes TDP-43 dysfunction. In this review, I propose a model that is centered on the loss-of-function mechanism whereby TDP-43 plays its role in neurodegeneration. I will highlight the evidence in the current literature that is consistent with this model and the evidence that is still needed from future experiments to test this model.

### A model for the loss of TDP-43 function as a central mechanism of pathogenesis in human disease

**Figure 1:**
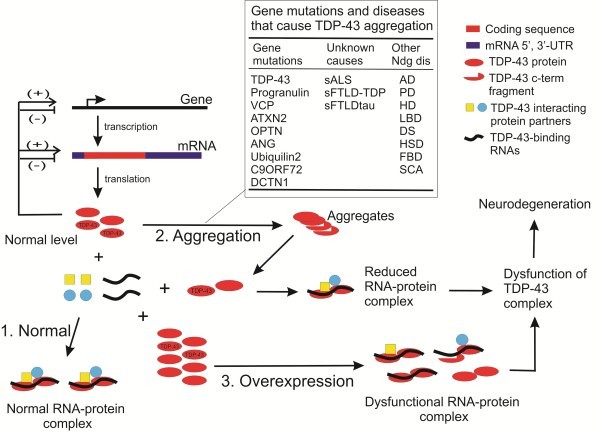
**Mechanisms that can cause TDP-43 dysfunction in ALS, FTLD and other neurodegenerative conditions.** AD means Alzheimer’s disease, PD Parkinson’s disease, HD Huntington’s disease, LBD Lewy body dementia, DS Down syndrome, HSD hippocampal sclerosis dementia, FBD familial British dementia, and SCA spinal cerebellar ataxia. See the section subtitled “A model for the loss of TDP-43 function as a central mechanism of pathogenesis in human disease” for a detailed description of this diagram.

The TDP-43 protein is normally expressed through transcription and translation, and once produced, it regulates its own expression by a feedback mechanism, i.e., upregulating its own expression when the protein level is too low and inhibiting its expression when the protein level is too high [15,22-27]. By this auto-regulatory mechanism, the intracellular level of TDP-43 is maintained within a narrow range (Figure 1, #1 normal). This tightly maintained TDP-43 level may be important because TDP-43 functions in multiprotein/RNA complexes [18-21], where a proper structure and function of the complex requires a certain stoichiometric ratio between TDP-43 and its protein and RNA partners (Figure 1, #1 normal). Such a requirement is not unique to TDP-43 complexes as it has been demonstrated in other protein-RNA or protein complexes. For example, in the primary micro RNA (pri-miRNA) processing Drosha complex, overexpression of one subunit DGCR8 leads to an inhibition in the processing activity [46]. As another example, in the kinesin-2 heterotrimeric complex that drives the antegrade transport of late endosomes and lysosomes, overexpression of one subunit KAP3 inhibited the transport similar to the KAP3 knockdown [47].

In the disease situation, conditions in patients’ cells become conducive for TDP-43 aggregation. For example, TDP-43 mutants and its C-terminal fragments associated with ALS and FTLD have enhanced aggregation propensity [48-51], and therefore, can drive TDP-43 aggregation. The aggregation can lead to a reduction in the pool of TDP-43 that can be incorporated into the TDP-43 protein/RNA complexes (Figure 1, #2 aggregation), thereby reducing the complex function and leading to neurodegeneration.

In model systems where TDP-43 is overexpressed (Figure 1, #3), the function of TDP-43 can be inhibited because an oversupply of exogenous TDP-43 mismatches with a limited supply of its endogenous interacting protein/RNA partners, resulting in the formation of incomplete and dysfunctional complexes. Below I highlight the evidence in the current literature that is consistent with this model and the future experiments that are need to test this model.

### TDP-43 performs functions of vital importance, but the consequence of its dysfunction in neurodegeneration remains unclear

A crucial piece of evidence for a loss-of-function mechanism would be demonstration that a loss of TDP-43 function can cause neurodegeneration. This has not yet been experimentally achieved in a convincing manner, particularly in mammalian species. Knockouts in rodents cause early embryonic lethality [25-27,36]. Inducible knockout in adult mice causes a rapid loss of fat tissue and lethality [36]. These results have not been informative as to the consequences of TDP-43 dysfunction in the nervous system. Nevertheless, the severity of the phenotype in the knockout models suggests a critical functional importance of TDP-43 in the health and survival of mammalian cells. Indeed, the conditional knockout of TDP-43 in mouse embryonic stem cells causes cell death [36]. Therefore, it is conceivable that TDP-43 function may also be vital for the survival and function of neurons. Supporting this notion are the experiments where TDP-43 knockdown causes morphological abnormalities and cell death in cultured neurons [50,52,53] and a large change in gene expression in cells of the CNS [15,16].

Experimental data from non-mammalian species have also been consistent with the critical functional importance of TDP-43. In *C. Elegans*, TDP-43 deletion mutants are viable, but show low fertility, slow growth and locomotor defects [44]. In Drosophila, TDP-43 knockout causes abortive embryonic development and lethality [30,31]. Although some escape the lethality and develop to adults, they display severe locomotor defects, premature death and abnormal neuronal morphology [30,31]. Evidence for progressive axonal degeneration and locomotor defects has also been reported in adult TDP-43 knockdown flies [32]. In zebrafish, TDP-43 knockdown during embryonic development causes selective defects in motor axonal growth and results in motor behavioral abnormalities [35]. These results do not conclusively demonstrate a role of TDP-43 dysfunction in neurodegeneration in ALS and FTLD, but do indicate that TDP-43 is important in the development and functioning of the nervous system, thus leaving open the possibility that TDP-43 dysfunction could play a role in neurodegeneration.

### How a loss of TDP-43 function explains the pathogenic mechanism of TDP-43 mutants

Mutations in TDP-43 cause motor neuron degeneration and ALS [28,29]. The overwhelming majority of the mutations are located in the C-terminal glycine-rich domain [12], which is unstructured and responsible for interactions with other proteins [17,21,54]. How mutant TDP-43 causes neurodegeneration is not known. Overexpression models support a gain of function, but the reliance of overexpression to elicit neurodegenerative phenotypes risks over-interpretation. A lack of convincing evidence that TDP-43 levels are elevated in human disease leaves open the question of whether the results from the overexpression models are relevant for the human disease.

While there is room for doubt for the gain-of-function mechanism, evidence for the loss-of-function mechanism is also weak, primarily because few experiments have generated data directly relevant to this question, especially in mammalian systems. Nevertheless, reasonable scenarios for this mechanism can be formulated based on the current, albeit fragmented and incomplete, experimental literature. First, wild-type TDP-43 is an aggregation-prone protein and mutant TDP-43 is even more so [48,51,55]. Therefore, TDP-43 mutants can initiate and drive protein aggregation, leading to TDP-43 depletion from the cell nucleus, as has been observed in patients [1,2,56]. In addition, mutant TDP-43 may have an enhanced susceptibility for polypeptide fragmentation, which generates the patient-specific 25-kDa fragments [29,57]. These fragments have a high propensity for aggregation [50,55,58] and can coaggregate with wild-type TDP-43, thereby sequestering wild-type TDP-43 into the aggregates and depleting TDP-43 from the nucleus [50].

Second, the mutant may be functionally less active or inactive but may still retain its autoregulation capability. As a result, the overall TDP-43 level would be maintained but the function of TDP-43 would be reduced because the protein expressed from the mutant allele is dysfunctional. Some experimental data support this scenario. In mice, overexpression of mutant TDP-43 inhibited the expression of the endogenous TDP-43 to the same extent as wild type overexpression [23,37,38], suggesting that the disease-causing mutants retain their autoregulatory function. In Drosophila, wild-type TDP-43 is capable of promoting growth of dendrites and increasing the size of synaptic terminals at the neuromuscular junction. However, these activities are lost in the ALS-causing mutants [31,34], suggesting that the mutants have lost some of the wild-type functions.

Third, mutant TDP-43 may form defective TDP-43 protein/RNA complexes, thereby poisoning the function of the complex. In this capacity, the mutant TDP-43 can act dominant-negatively to inhibit the function of the wild-type allele. There is evidence that TDP-43 forms a homodimer [59] and that multiple TDP-43 molecules are incorporated into each complex [19]. Therefore, if a mutant TDP-43 molecule were capable of rendering dysfunction to the whole complex that contains both mutant and wild-type TDP-43 molecules, then the function of the wild-type allele would be inhibited.

These scenarios are consistent with a model where TDP-43 mutants cause a loss of TDP-43 function by a dominant negative mechanism. Notably, while the first scenario requires the formation of aggregates for cellular toxicity, the second and third scenarios make such a requirement unnecessary. Indeed, in both cellular and animal models, toxicity induced by mutant TDP-43 does not require its aggregation [33,37,39,60].

### How TDP-43 dysfunction could contribute to neurotoxicity from overexpression of either mutant or wild-type TDP-43 in model systems

The prevailing interpretation for the observation that overexpression of mutant TDP-43 causes neurodegeneration is that mutant TDP-43 exert its toxicity by a gain of function. However, these results are also consistent with a dominant-negative mechanism, as discussed above (also see Table 1). The dominant-negative model predicts that overexpression of the mutant in sufficient quantities will inhibit the function of the two endogenous wild-type alleles in the model systems.

A puzzling observation is that overexpression of wild-type TDP-43 causes similar neurotoxic phenotypes in model systems [23,33,35,37,38,40-43,60,61]. Because of the autoregulatory mechanism, overexpression of human wild-type TDP-43 leads to a suppression of the endogenous TDP-43 [23,24]. This has led to a proposal that a loss of the endogenous TDP-43 caused neurotoxicity [24]. While this proposal can reasonably explain the toxicity of the mutants on the premise that they are dysfunctional, the toxicity from the wild-type TDP-43 poses a problem because several studies have shown that the human wild-type TDP-43 gene can substitute the function of its homologue in species as distant as *Drosophila* and *C. Elegans*[30,44]. A more plausible explanation can be derived from the fact that TDP-43 functions in multiprotein/RNA complexes, whose function may depend on a certain stoichiometric composition of the different protein/RNA components. Overexpression of wild-type TDP-43 provides an amount of TDP-43 in excess of the other components that form the complexes, thereby sequestering those components into incomplete and dysfunctional complexes (Figure 1, #3 overexpression). Therefore, both overexpression of the mutants and the wild-type TDP-43 can cause neurodegeneration by dominant-negatively inhibiting the normal function of TDP-43 complexes so long as it interacts with two or more components in the complexes simultaneously and with near equal binding affinities.

While the above interpretation of the literature remains to be confirmed by further experimentation, some of the predictions from this loss-of-function/dominant-negative hypothesis are supported by observations in the current literature. First, overexpression of mutant should be more potent in causing neurodegeneration than overexpression of the wild type, which has been the case in several overexpression models [35,40,60,61]. Although this finding is not inconsistent with the gain-of-function mechanism, the result can also be explained readily by the dominant-negative mechanism outlined above. Overexpression of mutants can inhibit normal TDP-43 function by three mechanisms: (1) displacing the endogenous TDP-43 through the autoregulation mechanism, (2) inserting itself into the TDP-43 complexes in the place of the wild-type protein, and (3) forming dysfunctional complexes by disruption of the stoichiometry between TDP-43 and other protein/RNA components. In contrast, overexpression of the wild-type TDP-43 can inhibit TDP-43 function only through the third mechanism because unlike the mutant protein, it has full function. Therefore, to inhibit TDP-43 function to the same degree, a higher level of expression will be required for the wild-type TDP-43 than the mutant.

Second, if the dominant-negative hypothesis is correct, overexpression and knockout or knockdown of the gene can cause similar phenotypes. Currently, data from mammalian species is lacking to address this point. However, evidence can be drawn from other species. For example, overexpression of either mutant or the wild-type TDP-43 in Drosophila motor neurons causes progressive locomotor defects and a shortening of lifespan [33]. These phenotypes are similar to those caused by TDP-43 knockdown [33]. As another example, expression of human TDP-43 mutants but not the wild type in zebrafish embryos compromised motor axonal growth and caused locomotor defects. Similarly as in flies, knocking down the endogenous TDP-43 caused the same phenotypes [35]. Importantly, the phenotypes in the knockdown fish are rescued by the expression of human wild-type TDP-43 but not the mutants. These results are consistent with the view that the ALS-relevant TDP-43 mutants are dysfunctional and are capable of inhibiting TDP-43 function in a dominant negative manner.

Third, the loss-of-function/dominant-negative hypothesis predicts that ALS-causing mutants should be loss-of-function alleles. As discussed above, the observations that the mutants lost their ability to stimulate the growth of dendrites and axons in flies [31,34,35] and their inability to rescue phenotypes from TDP-43 knockdown in zebrafish [35] supports the loss-of-function proposition. However, key evidence from mammalian species remains to be produced.

While the case for a loss of function by a dominant-negative mechanism can be argued for, it may be overly simplistic to argue that a gain of function does not contribute to the phenotypes caused by TDP-43 overexpression in the model systems. Some evidence indicate that TDP-43 is capable of causing cellular toxicity by a gain of function under ectopic and overexpressed conditions. For example, TDP-43 causes toxicity in yeast, which does not possess an endogenous TDP-43 homologue [62]. Similarly, TDP-43 is not essential in *C. Elegans*, yet overexpression of human TDP-43 can still cause toxicity that is not observed in knockouts [44,61,63,64]. Therefore, in model systems where TDP-43 performs vital functions, phenotypes caused by TDP-43 overexpression are likely derived from both an interference of endogenous TDP-43 function and a gain of function. Given the complexity in the protein/RNA interaction networks of TDP-43, perhaps this would not be surprising. Overexpression is likely to generate new aberrant interactions as well as to disrupt the authentic interactions that are vital for the cell. Therefore, disentangling these effects will be complex in the overexpression models.

### What is the role of wild type TDP-43 in human neurodegeneration

While the case for a loss of function in the TDP-43 mutants and in the overexpression model systems can be made, can the loss-of-function mechanism play a role in patients where TDP-43 is not mutated and not overexpressed? This is an important question because the vast majority of patients with ALS and FTLD-TDP do not have TDP-43 mutations. The answer to this question is yes because even though the primary trigger of the degenerative process lies not in TDP-43 but elsewhere, the same kind of TDP-43 aggregation and nuclear clearance is observed in the CNS of these patients [1,2,45] (Figure 1). The loss-of-function/dominant-negative model will predict that the nuclear clearance and the cytoplasmic aggregation of TDP-43 are probably a significant contributor to neurodegeneration by causing a loss of TDP-43 function. However, the experimental data for testing this prediction is scarce. In Drosophila and zebrafish, knockout or knockdown of TDP-43 produced similar neurodegenerative phenotypes [33,35]. However, further analysis is needed to differentiate the effects of TDP-43 dysfunction on neurodegeneration from those on neurodevelopment, and the relevance of these observations to human neurodegeneration remains to be established. A mammalian model with TDP-43 dysfunction in the mature CNS is urgently needed to understand the effects from a loss of TDP-43-function.

Based on the loss-of-function/dominant-negative hypothesis outlined above, what triggers TDP-43 aggregation will be one of the most intriguing and important questions in understanding the pathogenic mechanisms in ALS and FTLD. Recent investigations have shown that multiple causes can trigger secondary TDP-43 aggregation and nuclear clearance. These causes can be classified into several categories: (1) Gene mutations that enhance the mutant protein aggregation propensity and cause ALS-FTLD with TDP-43 aggregation. Examples in this category include VCP, optineurin, dynactin, ataxin 2 and ubiquilin 2. All the mutant proteins form aggregates and some form coaggregates with wild-type TDP-43 [9,65-69]. The mechanism whereby these mutants cause TDP-43 aggregation is not understood. One possibility is that the aggregation of these proteins weakens the capacity of cellular proteostasis [70], which creates an environment conducive for aggregation-prone proteins such as TDP-43 to aggregate. Some of the proteins such as VCP and ubiquilin may be involved in TDP-43 degradation [71,72]. Therefore, mutations in these proteins may directly alter the TDP-43 economy and cause TDP-43 aggregation. (2) Gene mutations that cause ALS and FTLD with TDP-43 aggregation, but the mutant proteins are not involved in protein aggregation themselves. Examples in this category include progranulin, angiogenin and C9ORF72 [1,11,73,74]. At present, it is not known how these mutations lead to TDP-43 aggregation. (3) Traumatic brain injury that lead to ALS-FTLD without gene mutations. Repetitive traumatic brain injury has been shown to be associated with ALS and FTLD with intracellular TDP-43 aggregation [75,76]. (4) Other neurodegenerative diseases that are not ALS-FTLD but trigger secondary TDP-43 aggregation. Examples of this category include some of the most common neurodegenerative diseases such as Alzheimer’s disease, Parkinson’s disease and numerous others [8,77,78] (Figure 1). Aggregation of TDP-43 in these cases may also be attributed to a disruption of proteostasis environment due to the aggregation of other proteins, although direct experimental evidence for this hypothesis is not yet in existence. (5) Unknown causes in sporadic ALS and FTLD cases. Some of the speculated causes include genetic predisposition in combination with environmental stress, e.g. environmental toxins, trauma and high physical activity [79-82].

Recent studies have suggested that a redistribution of TDP-43 to the cytoplasm may be a precursor to TDP-43 aggregation. In ALS and FTLD patients, some neurons show an increase in cytoplasmic TDP-43 immunoreactivity with diffused or granular appearance, which may represent an early stage of TDP-43 aggregation [83-86]. The cause for the cytoplasmic redistribution is not clear. However, a recent study demonstrate that a single traumatic brain injury can be followed by a persistent increase in the cytoplasmic levels of TDP-43 [87], suggesting that injuries to the CNS can be an initial trigger for increased levels of cytoplasmic TDP-43. In model systems, the redistribution of TDP-43 can be triggered by various stresses, including neuronal injury [88-90], overexpression of disease-associated mutant TDP-43 and VCP [91-93], oxidative stress [93,94] and proteasome inhibition [53]. The functional consequence of the cytoplasmic localization of TDP-43 will require further characterization. Nevertheless, some studies suggest that the cytoplasm-localized TDP-43 is recruited to stress granules before being transformed into aggregates that can persist independent of stress granules [93-95]. Another study demonstrated that a modest knockdown of TDP-43 exacerbated, rather than alleviated, cell death that is induced by proteasome inhibition and associated with TDP-43 cytoplasmic translocation [53], suggesting that any toxicity that might be associated with TDP-43 cytoplasmic translocation is derived from a loss of TDP-43 function. These data are consistent with the hypothesis that an increased cytoplasmic level of TDP-43, which follows the initial cellular stress, can lead to TDP-43 aggregation and nuclear depletion.

### Therapeutic implications from the dominant-negative model

Discussion on therapeutic implication based on the loss-of-function hypothesis may be premature since the hypothesis remains to be tested. However, such an exercise may be helpful for illustration of the critical importance for a resolution of this question. In the case of a gain of function, strategies that reduce the function should be effective. This may be achieved by lowering the protein levels through an inhibition of its synthesis or a stimulation of its degradation. If the toxic activity is known, strategies that inhibit the specific toxic activity may also be effective. In the case of a loss of function, on the other hand, strategies that increase the function should be effective. This may be achieved by increasing expression and stability of the protein, or stimulating its activity.

The therapeutic strategy for the dominant negative mechanism differs from both purely gain- or loss-of-function mechanisms and will be most challenging. We cannot simply increase the level of TDP-43 because uncontrolled increase of TDP-43 may inhibit the function of TDP-43 rather than improving it. High levels of TDP-43 could also further accelerate its aggregation and produce aberrant interactions with other proteins and RNA. Moreover, we do not understand why TDP-43 stays in the cytoplasm and becomes depleted from the nucleus in the disease. Therefore, it is not clear whether a simple increase of TDP-43 will replenish its level in the nucleus. In the case of mutant TDP-43, allele-specific inhibition of the mutant TDP-43 may be helpful but may not be sufficient to compensate for the lost function of the mutant allele. If the hypothesis that TDP-43 aggregation drives nuclear depletion of the TDP-43 is correct, preventing or reversing the aggregation may be a rational and safe approach to mitigate the loss of TDP-43 function. To achieve this, we need to understand how TDP-43 aggregation is triggered and propagated. We also need to understand the TDP-43 aggregation process at molecular and structural levels. Alternatively, strategies that enhance the function of TDP-43 without resorting to increase the protein level, or retain TDP-43 in the nucleus may also be effective.

## Conclusions

TDP-43 aggregation and nuclear depletion have been observed widely in neurodegenerative diseases. The role of TDP-43 in neurodegeneration remains to be defined. Chief among the questions is whether a gain of function, a loss of function or a dominant-negative mechanism is responsible for neurotoxicity. The answer to this question is of critical importance because it guides the future direction of research and sets the foundation for therapeutic strategies. Current experimental data from model systems has been predominantly invoked to support the gain-of-function mechanism. However, a careful review of the data suggests that a loss of TDP-43 function caused by its mutations, its aggregation and nuclear depletion, and the inhibition of TDP-43 function by a dominant-negative mechanism in the overexpression models, are at least as plausible as the gain-of-function theory, if not more so. Therefore, in our future research, we need to gain a more detailed understanding of the normal function of TDP-43, particularly in the cells of the CNS. We need models of loss of TDP-43 function in the CNS, particularly in mammalian species, to understand the consequence of TDP-43 dysfunction. In such a pursuit, models with a partial loss of TDP-43 function may be especially desirable because in humans, it is unlikely that the TDP-43 function is totally lost. We need evidence from human diseases to determine whether the conditions are more in tune with a gain or a loss of TDP-43 function. Lastly, we need to design strategies to address the difficult problem of how to restore the normal levels of TDP-43 function as a therapy.

## Competing interests

The authors declared that they have no competing interests.

## Authors’ contributions

ZX conceived the ideas and wrote the manuscript.Notes after the proof After the proof of this article, James Shen and colleagues demonstrated that deletion of TDP-43 in motor neurons led to a loss of motor neuron in mice (J Biol Chem, in press), thus indicating that, similar to other cell types, TDP-43 function is also required for motor neuron survival.
